# Polyinosinic: polycytidylic acid induced inflammation enhances while lipopolysaccharide diminishes alloimmunity to platelet transfusion in mice

**DOI:** 10.3389/fimmu.2023.1281130

**Published:** 2023-12-11

**Authors:** Johnson Q. Tran, Marcus O. Muench, Betty Gaillard, Orsolya Darst, Mary M. Tomayko, Rachael P. Jackman

**Affiliations:** ^1^ Vitalant Research Institute, San Francisco, CA, United States; ^2^ Department of Laboratory Medicine, University of California, San Francisco, San Francisco, CA, United States; ^3^ Department of Dermatology, Yale University School of Medicine, New Haven, CT, United States; ^4^ Department of Pathology, Yale University School of Medicine, New Haven, CT, United States

**Keywords:** platelets, alloimmunization, underlying health, inflammation, poly(I:C), lipopolysaccharide, alloantibodies

## Abstract

**Introduction:**

Alloimmune responses against platelet antigens, which dominantly target the major histocompatibility complex (MHC), can cause adverse reactions to subsequent platelet transfusions, platelet refractoriness, or rejection of future transplants. Platelet transfusion recipients include individuals experiencing severe bacterial or viral infections, and how their underlying health modulates platelet alloimmunity is not well understood.

**Methods:**

This study investigated the effect of underlying inflammation on platelet alloimmunization by modelling viral-like inflammation with polyinosinic-polycytidylic acid (poly(I:C)) or gram-negative bacterial infection with lipopolysaccharide (LPS), hypothesizing that underlying inflammation enhances alloimmunization. Mice were pretreated with poly(I:C), LPS, or nothing, then transfused with non-leukoreduced or leukoreduced platelets. Alloantibodies and allogeneic MHC-specific B cell (allo-B cell) responses were evaluated two weeks later. Rare populations of allo-B cells were identified using MHC tetramers.

**Results:**

Relative to platelet transfusion alone, prior exposure to poly(I:C) increased the alloantibody response to allogeneic platelet transfusion whereas prior exposure to LPS diminished responses. Prior exposure to poly(I:C) had equivalent, if not moderately diminished, allo-B cell responses relative to platelet transfusion alone and exhibited more robust allo-B cell memory development. Conversely, prior exposure to LPS resulted in diminished allo-B cell frequency, activation, antigen experience, and germinal center formation and altered memory B cell responses.

**Discussion:**

In conclusion, not all inflammatory environments enhance bystander responses and prior inflammation mediated by LPS on gram-negative bacteria may in fact curtail platelet alloimmunization.

## 1 Introduction

Transfusion of platelet concentrates is a critically important, life-saving procedure to treat thrombocytopenia in the context of congenital disorders, surgery, hemorrhagic trauma, and cancer therapy and continues to be developed for novel applications in regenerative medicine (1–4). A major complication emerges, however, when transfusion of allogeneic platelets is recognized by the recipient immune system as foreign, which can progress to alloimmunization against a range of alloantigens, the dominant being the major histocompatibility complex (MHC) antigens expressed on platelets and white blood cells (WBCs) (5). Long-term adverse post-transfusion sequelae of alloimmunization include platelet refractoriness to subsequent platelet transfusion (class I MHC alloimmunization) and rejection of future solid organ or hematopoietic stem cell transplants (class I and II MHC alloimmunization) in some patients (6–11).

Several practices have been adopted to mitigate the risk of alloimmunization following platelet transfusion such as reducing the immunogenicity of blood products. Modern leukoreduction practices, which filter the highly immunogenic WBCs from blood products, have decreased alloimmunization risk (12–18), however, recent estimates suggest alloantibody generation still occurs in ~4-18% of platelet recipients, where the responses are largely directed against MHC antigens on platelets and residual leukocytes (19–22). A potential complementary approach to mitigate alloimmunization risk may be to identify which recipients are at greatest risk of alloimmunization due to genetic factors or underlying health to better manage care. Transfusion recipients normally receive blood products because of a medical condition, and these conditions can create inflammatory or immunotolerant environments for blood-derived alloantigens. Advances have been made to understand the effects of underlying health on red blood cell (RBC) transfusions (23–27), but there is a dearth of research on its effects on alloimmunization to platelets.

Estimates suggest 10-14% of patients admitted to the hospital for severe bacterial or viral infections received RBC transfusions and ~6% received platelet transfusion as a component of their care (27–29). Bacterial and viral infections are synonymous with inflammation, as microorganism derived pathogen-associated molecular patterns (PAMPs) are quintessential stimulators of innate immunity (30). In a conventional immune response, adaptive immunity responds in an antigen-specific manner (31). However, ongoing infection and inflammation can elicit bystander activation where off-target adaptive immunity is activated in an antigen-independent manner due to a heightened inflammatory cytokine milieu (32, 33). Virus-mimetics such as polyinosinic-polycytidylic acid (poly(I:C)) and the bacterial component lipopolysaccharide (LPS) from gram-negative bacteria have been shown to induce bystander activation when directly injected into mice (34, 35). Therefore, patients admitted to the hospital for infection may sustain a reduced threshold for immune activation and consequently potentiate alloimmunization to subsequent exposure to platelet alloantigens during transfusion.

Conducting studies on the effect of underlying health on platelet alloimmunization is challenging clinically due to confounding factors such as the diversity of the patient population and the variability of the type and amount of blood products they receive. Furthermore, alloantigen-specific B cells (allo-B cells) are a rare population and in the presence of inflammatory signals may be further masked by non-specific activation. To address these issues, this study used murine models and MHC tetramers to track allo-B cell responses. The impact of underlying inflammation on platelet alloimmunization was evaluated using poly(I:C) to mimic a viral infection or LPS to mimic a bacterial infection prior to platelet transfusion with the hypothesis that underlying inflammation enhances alloimmunization.

## 2 Materials and methods

### 2.1 Mice

Female BALB/cJ (BALB/c) recipient mice, aged 9 weeks, male and female C57Bl/6J (B6) donor mice, aged 2-12 months, and male and female B6;129P2-β2m^tm1Unc^/J mice (β2M-KO) were received from The Jackson Laboratory, and/or bred in-house, and maintained in a specific pathogen free vivarium at Vitalant Research Institute (San Francisco, CA) (36). Mice acclimated for at least two weeks prior to use. Research was approved by the Institutional Animal Care and Use Committee at Labcorp Early Developmental Laboratories Inc. (San Carlos, CA) under Animal Welfare Assurance A3367-01.

### 2.2 Poly(I:C), LPS, and platelets

**Figure 1:**
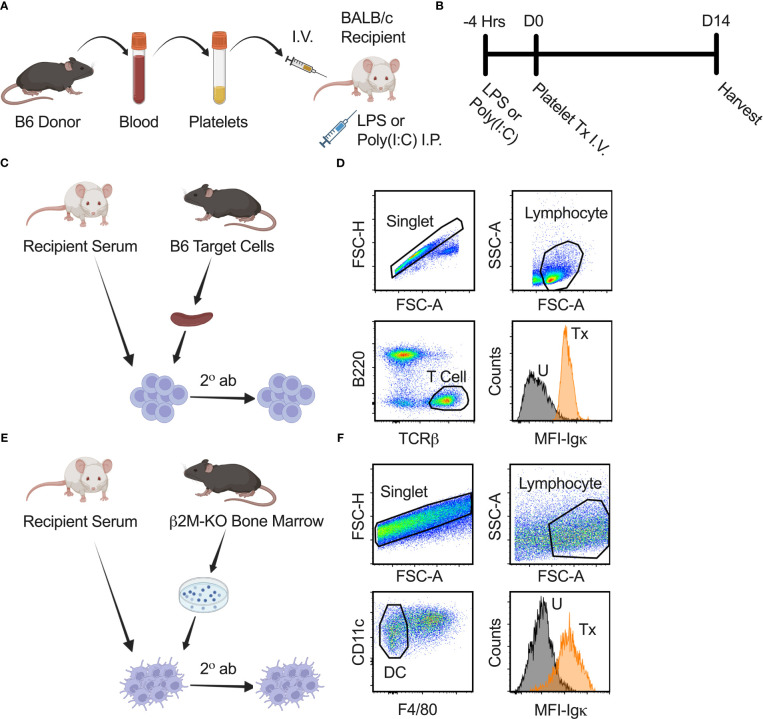
Experimental strategy and anti-class I and II MHC antibody screen. **(A, B)** Schematic diagram modelling the effect of underlying inflammation on platelet alloimmunization. B6 donor platelet was administered I.V. into recipient BALB/c mice on day zero (D0). Four hours prior (-4 Hrs) to platelet transfusion (Tx), LPS or poly(I:C) was injected into recipient BALB/c mice by intraperitoneal (I.P.) route to model underlying inflammation. Blood for serum, spleen, and lymph nodes from recipient mice were harvested on day fourteen (D14) after transfusion for analysis. **(C)** Schematic diagram of anti-class I MHC antibody screen. Serum from recipient BALB/c mice was used as a primary stain on B6 splenocytes, followed by a secondary antibody (2° ab) stain with a cocktail of α-Igκ, α-IgM, α-IgG1, α-IgG2a, α-IgG2b, and α-IgG3 antibodies to detect total and isotype-specific alloantibodies by flow cytometry. **(D)** Cells were gated on singlet, lymphocyte, and then T cells (B220^-^/TCRβ^+^) to identify bound alloantibodies on a non-Igκ, non-class II MHC population that expresses class I MHC. α-Igκ only shown as illustration. Median fluorescence intensity (MFI) of total α-Igκ alloantibodies for untreated (U) and with transfusion (Tx) sample are shown. **(E)** Schematic diagram of anti-class II MHC antibody screen. Protocol was similar to anti-class I MHC antibody screen except cultured dendritic cells (DC) from the bone marrow of β2M-KO mice on the B6 background were used as target cells. **(F)** Cells were gated on singlet, lymphocyte, and then dendritic cells (CD11c^+^/F4/80^-^) to identify bound alloantibodies on non-class I MHC population that expresses class II MHC.

Poly(I:C) (Innaxon) was prepared at 500 μg/mL and LPS (Sigma) at 125 μg/mL in sterile endotoxin free saline (Innaxon). Recipient BALB/c mice received 200 μL of poly(I:C) or LPS by intraperitoneal route four hours prior to platelet transfusion (+/- 15 minutes) (**Figure 1**). Controls were given poly(I:C) only, LPS only, transfusion (Tx) only, or no treatment.

**Figure 2:**
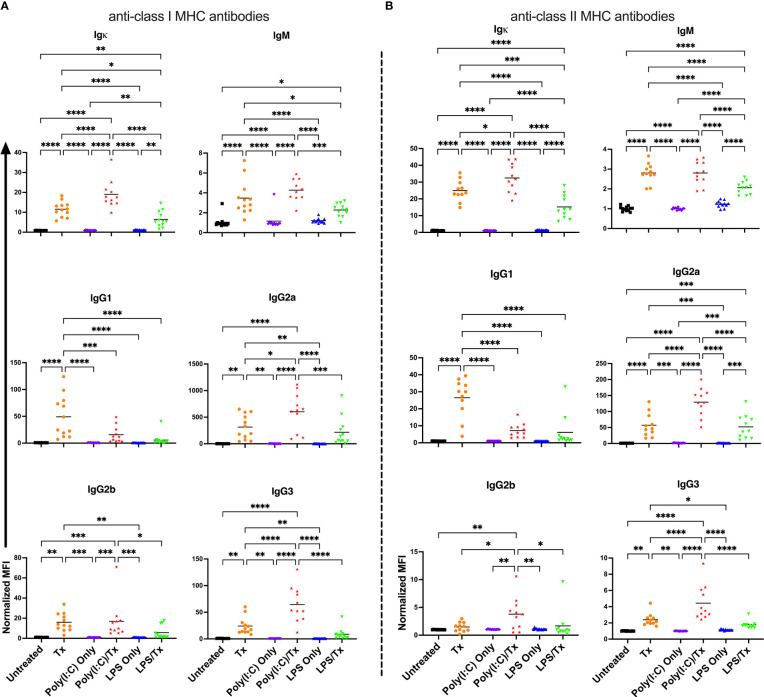
Poly(I:C) increases and LPS decreases the alloantibody response to non-leukoreduced platelet transfusion. **(A)** Total and isotype-specific anti-class I MHC antibody and **(B)** anti-class II MHC antibody responses at 14 days post non-leukoreduced platelet transfusion. For IgG1 for anti-class I MHC antibody (A), 100 was added to the raw values prior to normalization to adjust for negative values. Normalized MFI in mice that were untreated (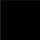), received transfusion alone (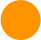), poly(I:C) alone (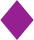), poly(I:C) followed by transfusion (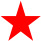), LPS alone (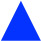), or LPS followed by transfusion (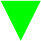). (p)<0.05 (*), p<0.01 (**), p<0.001 (***), and p<0.0001 (****).

**Figure 3:**
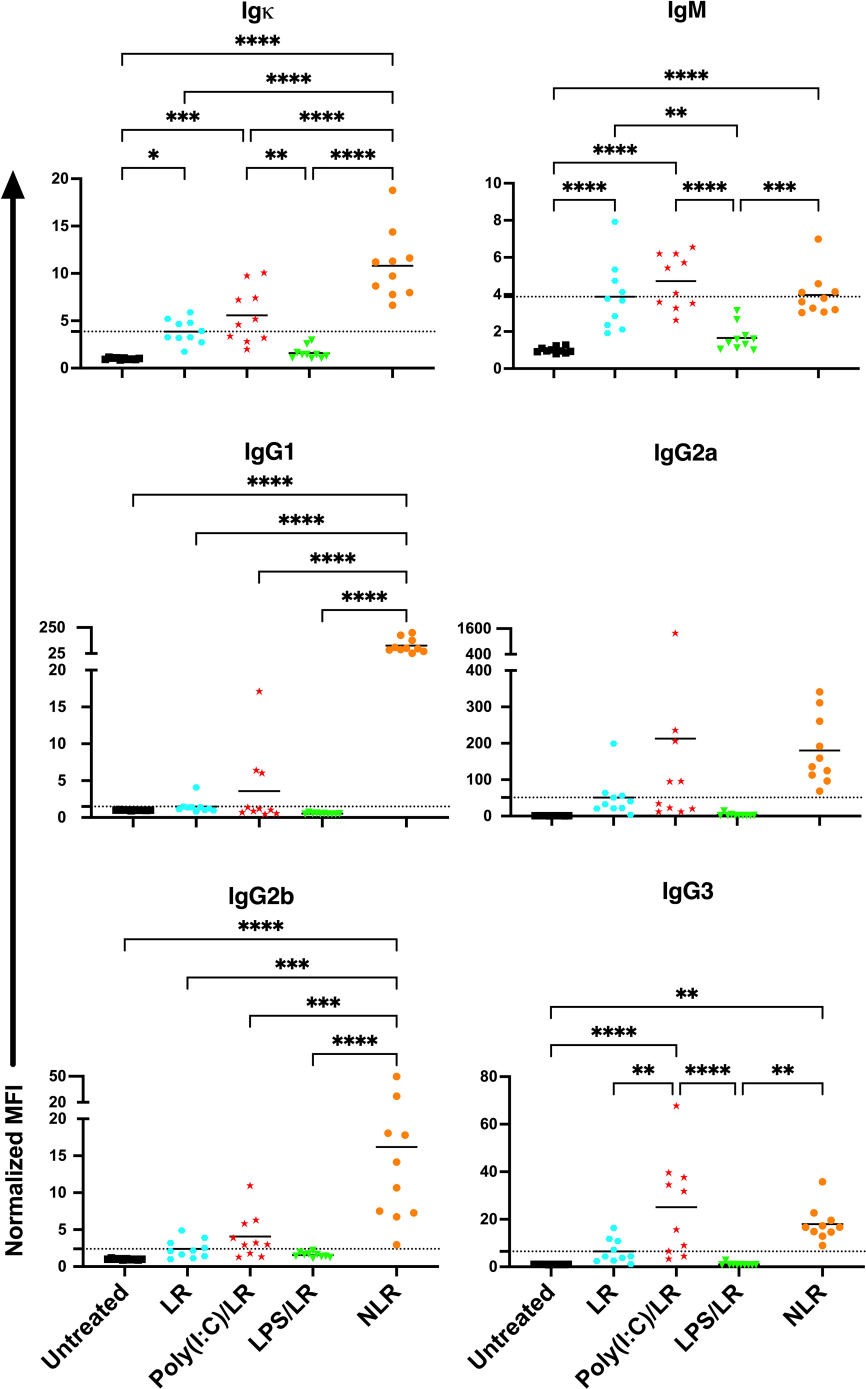
The effects of underlying inflammation on leukoreduced platelet transfusion are consistent with non- leukoreduced platelet transfusion, although the magnitude of the responses is smaller. Poly(I:C) increases and LPS decreases the alloantibody response to leukoreduced platelets. Normalized MFI in mice that were untreated (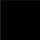), received leukoreduced (LR) platelet transfusion alone (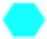), poly(I:C) followed by leukoreduced transfusion (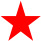), LPS followed by leukoreduced transfusion (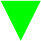), and non-leukoreduced (NLR) platelet transfusion alone (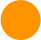). 10 mice per group in a single experiment. For IgG1 and IgG3, 200 was added to the raw values prior to normalization to adjust for negative values. (p)<0.05 (*), p<0.01 (**), p<0.001 (***), and p<0.0001 (****). Tick line represents mean of the group that received a leukoreduced transfusion only.

**Figure 4:**
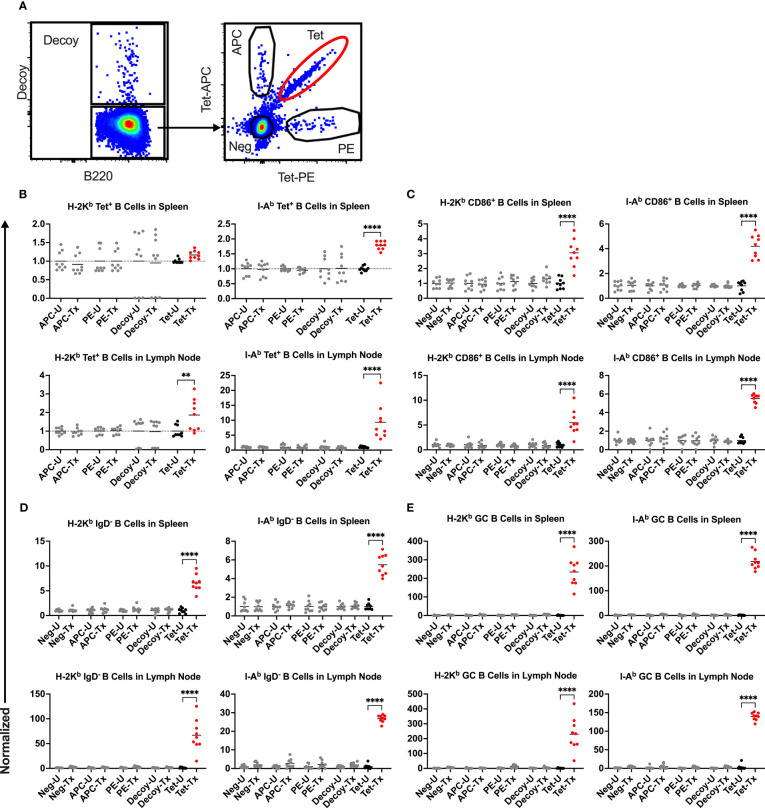
Platelet transfusion specifically induces allo-B cell responses. **(A)** B cell subsets of different specificities were examined for responses in untreated mice (U) and after platelet transfusion (Tx), including B cells specific for the MHC tetramer (Tet, allo-B cells), syngeneic decoy (Decoy), single fluorophore (APC or PE), or the remaining B cell population (Neg). Normalized data shows the frequency of **(B)** the respective B cell subset out of total B cells, **(C)** CD86^+^ activated B cells of the respective B cell subset (see **Figure 6** for gating), **(D)** IgD^-^ antigen-experienced B cells of the respective B cell subset (see **Figure 7** for gating), and **(E)** CD95^+^/CD38^-^ germinal center (GC) B cells of the respective B cell subset (see **Figure 8** for gating). **(B–E)** Results for splenic (top) and lymph node (bottom) cells were stained with MHC class I (left) and MHC class II (right) tetramers. Normalization was achieved by taking the average of the respective untreated group. Tick line falls on mean of untreated group of Neg subset. p<0.01 (**) and p<0.0001 (****).

**Figure 5:**
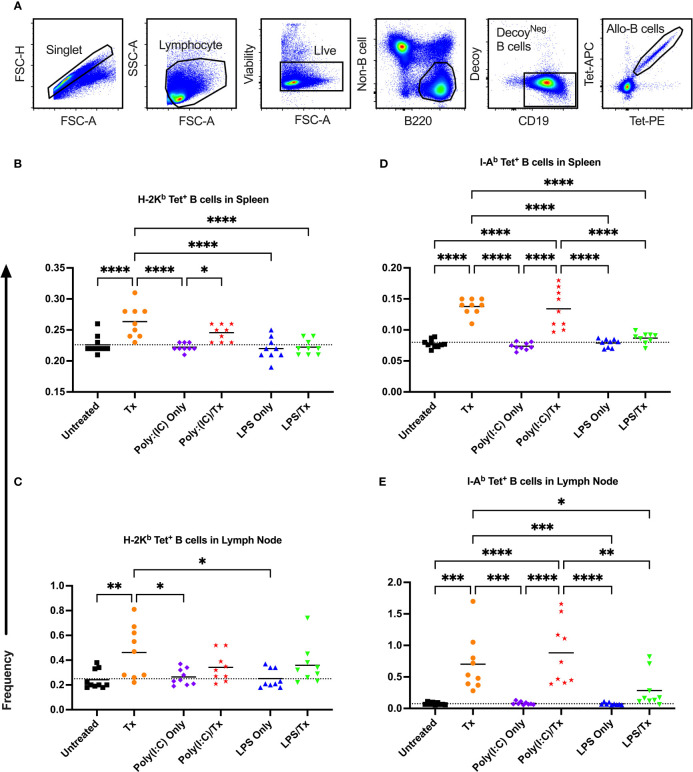
Transfusion-induced expansion of alloreactive B cells is impaired following exposure to LPS. Frequency of allo-B cells (out of total B cells without syngeneic decoy events) were identified with MHC tetramers by flow cytometry two weeks after platelet transfusion. **(A)** Cells were first gated on singlet, lymphocytes, live cells, non-B cell^-^/B220^+^ cells, and CD19^+^/syngeneic decoy^-^ B cells, then evaluated for tetramer staining. A two fluorophore (APC and PE)-one MHC tetramer strategy was used with allo-B cells identified as double positive tetramer (tet) events. **(B–E)** Shown is allo-B cell frequency in mice that were untreated (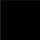), received transfusion alone (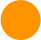), poly(I:C) alone (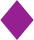), poly(I:C) followed by transfusion (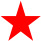), LPS alone (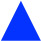), or LPS followed by transfusion (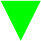). Splenic and lymph node cells were stained with MHC class I **(B, C)** and MHC class II **(D, E)** tetramers. Tick line represents mean of untreated group. (p)<0.05 (*), p<0.01 (**), p<0.001 (***), and p<0.0001 (****).

**Figure 6:**
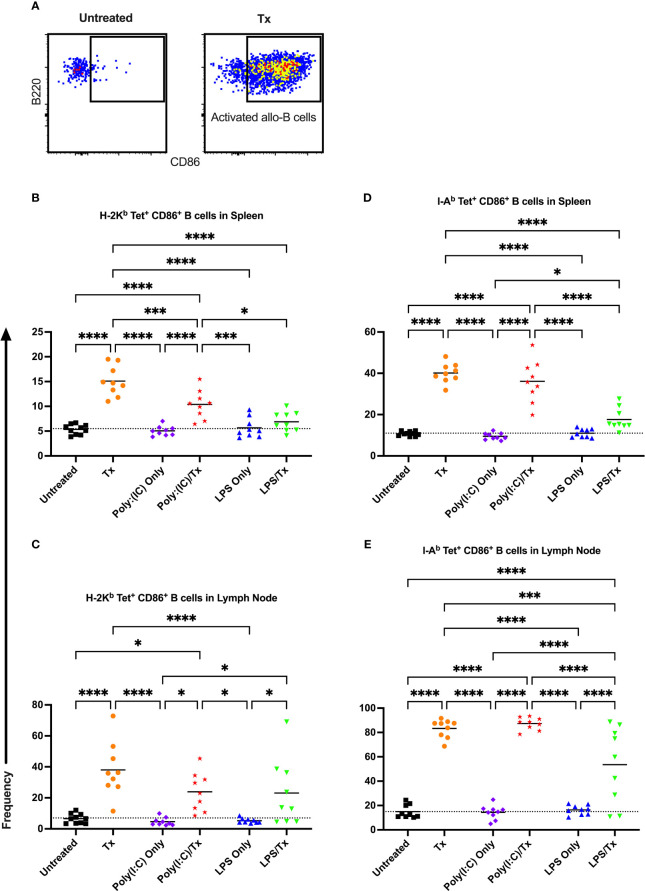
Transfusion-induced activation of alloreactive B cells is diminished following LPS exposure. **(A)** Frequency of activated CD86^+^ allo-B cells out of total allo-B cells was evaluated with representative examples shown of untreated and platelet transfused mice. **(B–E)** Shown is activated allo-B cell frequency in mice that were untreated (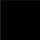), received transfusion alone (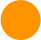), poly(I:C) alone (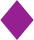), poly(I:C) followed by transfusion (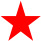), LPS alone (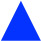), or LPS followed by transfusion (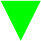). Splenic and lymph node cells were stained with MHC class I **(B, C)** and MHC class II **(D, E)** tetramers. Tick line represents mean of untreated group. (p)<0.05 (*), p<0.001 (***), and p<0.0001 (****).

**Figure 7:**
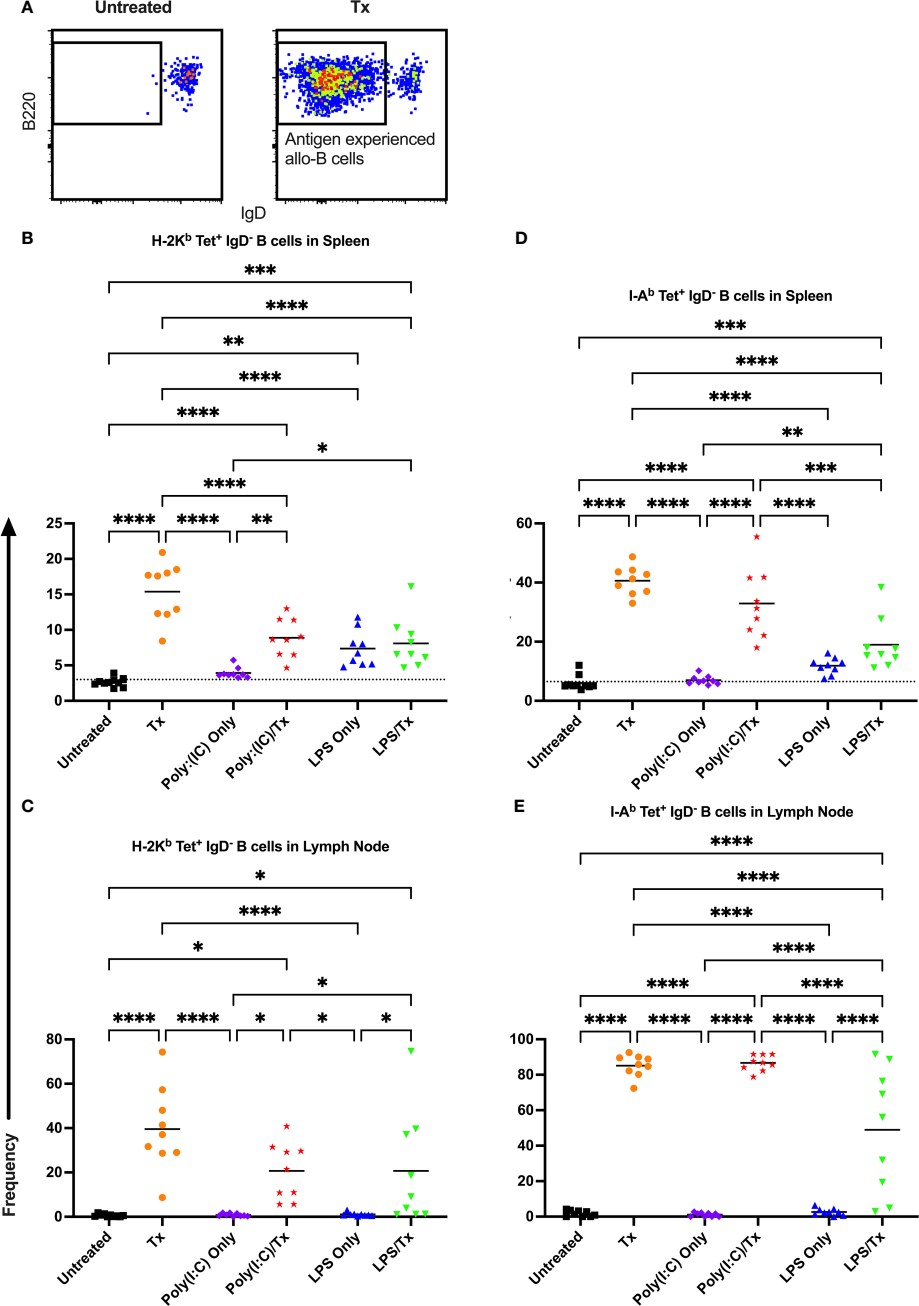
Transfusion-induced down-regulation of IgD is impaired with prior LPS exposure. **(A)** Frequency of antigen experienced IgD^-^ allo-B cells out of total allo-B cells was evaluated with representative examples shown of untreated and platelet transfused mice. **(B–E)** Shown is antigen experienced allo-B cell frequency in mice that were untreated (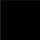), received transfusion alone (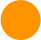), poly(I:C) alone (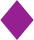), poly(I:C) followed by transfusion (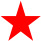), LPS alone (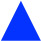), or LPS followed by transfusion (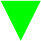). Splenic and lymph node cells were stained with MHC class I **(B, C)** and MHC class II **(D, E)** tetramers. Tick line represents mean of untreated group. (p)<0.05 (*), p<0.01 (**), p<0.001 (***), and p<0.0001 (****).

**Figure 8:**
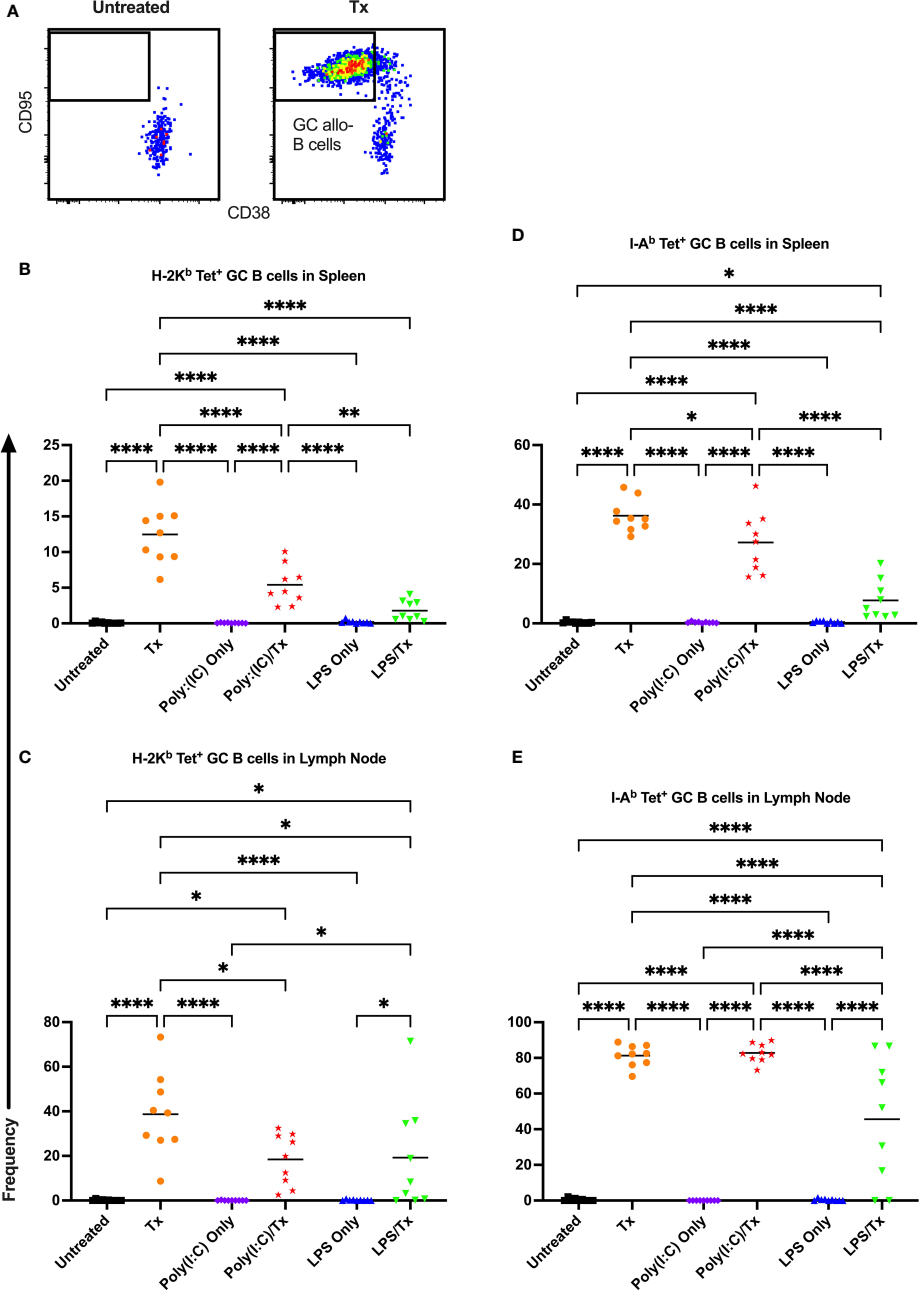
Germinal center response to transfusion is diminished with prior LPS exposure. **(A)** Frequency of germinal center (GC) CD95^+^/CD38^-^ allo-B cells out of total allo-B cells was evaluated with representative examples shown of untreated and platelet transfused mice. **(B–E)** Shown is germinal center allo-B cell frequency in mice that were untreated (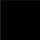), received transfusion alone (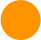), poly(I:C) alone (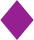), poly(I:C) followed by transfusion (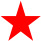), LPS alone (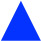), or LPS followed by transfusion (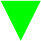). Splenic and lymph node cells were stained with MHC class I **(B, C)** and MHC class II **(D, E)** tetramers. (p)<0.05 (*), p<0.01 (**), and p<0.0001 (****).

**Figure 9:**
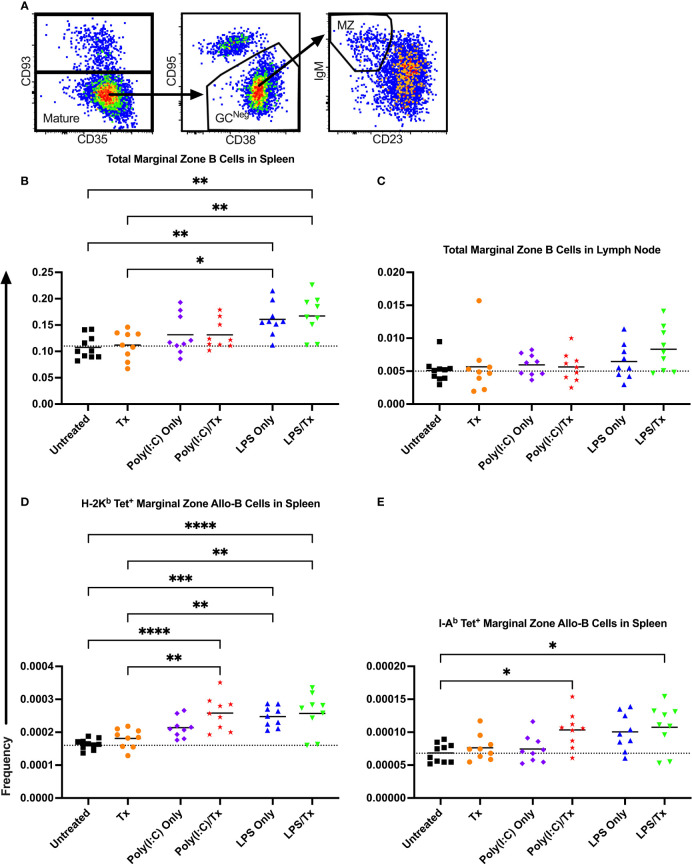
LPS non-specifically enriches for marginal zone B cells. **(A)** Representative gating selected for CD93^-^ mature B cells, CD95^-^/CD38^+^ non-GC (GC^Neg^) B cells, and IgM^+^/CD23^-^ marginal zone (MZ) B cells. Shown is frequency of total MZ B cells out of total B cells in spleen **(B)** and lymph node **(C)**. Frequency of marginal zone B cells within the allo-B cell subset population out of total B cells is also shown in the spleen which were stained with MHC class I **(D)** and MHC class II **(E)** tetramers. Mice were untreated (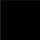), received transfusion alone (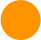), poly(I:C) alone (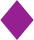), poly(I:C) followed by transfusion (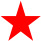), LPS alone (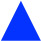), or LPS followed by transfusion (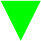). Tick line represents mean of untreated group. (p)<0.05 (*), p<0.01 (**), p<0.001 (***), and p<0.0001 (****).

**Figure 10:**
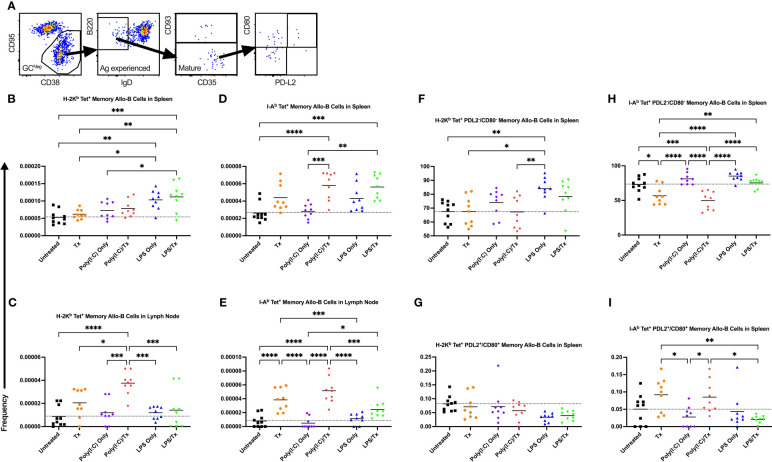
Underlying inflammation induced by poly(I:C) enriches for allo-B memory cells. **(A)** Gating strategy for allo-B memory cells selected for CD95^-^/CD38^+^ non-GC (GC^Neg^) B cells, B220^+^/IgD^-^ antigen (Ag) experienced B cells, and CD93^-^ mature B cells which represents total memory B cells. Total memory B cells were then selected for CD80^-^/PD-L2^-^ double negative and CD80^+^/PD-L2^+^ double positive subsets of allo-B memory cells. **(B–I)** Shown is frequency out of total B cells. Splenic and lymph node cells were stained with MHC class I **(B, C, F, G)** and MHC class II **(D, E, H, I)** tetramers. **(B–E)** Frequency of total allo-B memory cells. Frequency of CD80^-^/PD-L2^-^ double negative allo-B memory cells **(F, H)** and CD80^+^/PD-L2^+^ double positive allo-B memory cells **(G, I)**. Mice were untreated (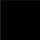), received transfusion alone (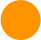), poly(I:C) alone (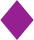), poly(I:C) followed by transfusion (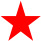), LPS alone (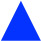), or LPS followed by transfusion (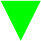). Tick line represents mean of untreated group. (p)<0.05 (*), p<0.01 (**), p<0.001 (***), and p<0.0001 (****).

Blood from B6 donor mice was collected by orbital enucleation under inhalation isoflurane anesthesia into 14% citrate phosphate dextrose adenine-1, and non-leukoreduced platelets were isolated from blood after gentle centrifugation and Ficoll separation as previously described (5, 17, 37–41). Complete blood counts (CBCs) were run on the HT-5 (Heska) according to manufacturer’s instructions. The non-leukoreduced platelet product had a mean of 3.98x10^8^ platelets/mL and 5.28x10^6^ WBCs/mL. Recipient BALB/c mice were given 100 μL of non-leukoreduced or leukoreduced platelets by lateral tail vein I.V. injection administered within four hours of collection. Non-leukoreduced platelets were used for **Figure 2**. Leukoreduced platelets were used for **Figure 3**. Non-leukoreduced platelets were used for **Figures 4**–**10**. Leukoreduced platelets were prepared as above except they were passed through a Pall Acrodisc WBC Syringe Filter right after collection. The leukoreduced platelet product had 5.67x10^8^ platelets/mL and WBCs were below the level of detection (<0.3x10^6^ cells/mL). Two weeks after transfusion, serum was collected to screen for alloantibodies, and the spleen and inguinal lymph nodes in some mice were harvested for cellular analysis.

### 2.3 Alloantibody screen

Total [Igκ represents ~95% of all murine B cells (42–47)] and isotype-specific anti-donor alloantibodies were evaluated in recipient serum by flow cytometry (**Figures 1C–F**). Blood was collected by orbital enucleation from recipient mice, allowed to clot in room temperature for 20-30 minutes, centrifuged to collect serum, and stored at -80°C. Thawed serum was screened against B6 target splenocytes for alloantibodies against class I MHC antigens or screened against cultured dendritic cells from β2M-KO mice for alloantibodies against class II MHC antigens. Dendritic cells were cultured from the bone marrow of β2M-KO mice for 11 days with granulocyte-macrophage colony-stimulating factor (GM-CSF) every 2 or 3 days and LPS 24 hours prior to collection. Target cells were blocked with F_c_ receptor block (F_c_R, 2.4G2, Becton Dickinson Biosciences, BD), stained with 1:16 diluted recipient serum, washed, and stained with a secondary antibody cocktail containing anti-mouse B220-Alexa Fluor (AF)700 (RA3-6B2), T cell receptor (TCR)β-peridinin-chlorophyll-protein (PerCP)-Cyanine (Cy)5.5 (H57-597), immunoglobulin (Ig)κ-allophycocyanin (APC)-Cy7 (RMK-45), IgG2b-phycoerythrin (PE; RMG2b-1), IgG2a-APC (RMG2a-62), IgG1-AF488 (RMG1-1), IgM-PE-Cy7 (RMM-1) (Biolegend), and IgG3-Brilliant Violet (BV)421 (R40-82; BD) for the anti-class I antibody screen. Anti-mouse F4/80-AF700 (BM8) and CD11c PerCP-Cy5.5 (N418) were used for the anti-class II antibody screen. Median fluorescence intensity (MFI) of Igκ and each isotype were normalized by dividing each sample value by the average of the untreated group.

### 2.4 B cell evaluations

Spleen and lymph nodes were homogenized by enzymatic digestion with 0.4 mg/mL collagenase P (Roche), 1.6 mg/mL dispase (Invitrogen), and 0.2 mg/mL DNase I (Roche) for 20 minutes at 37°C and passed through a 100 μM filter. Homogenates were subjected to red blood cell lysis, F_c_R block (2.4G2, BD), viability staining (BD Live/Dead Blue), and staining with antibody cocktail in Brilliant Stain Buffer (BD). Anti-mouse antibodies included: Sca-1-BV421 (D7), Sca-1-AF488 (D7), CD19-PerCP (6D5), CD138-BV711 (281–2), IgM-BV711 (RMM-1), CD11b-BV570 (M1/70), CD73-AF594 (TY/11.8), IgD-AF594 (11-26c.2a), and B220-BV650 (RA3-6B2) (Biolegend); CD86-Brilliant Blue (BB)700 (GL1), CD5-BV750 (53-7.3), CD80-BV480 (16-10A1), PD-L2-Brilliant Ultra Violet (BUV)737 (Ty25), CD95-BUV805 (Jo2), CD93-BV605 (AA4.1), CD35-BV650 (C12), CD38-BUV563 (90/CD38), IgM-BUV615 (II/41), CD23-BUV395 (B3B4) and IgD-BUV496 (AMS9.1) (BD); B220-AF532 (RM0063-9F14, Novus Biologicals); and CD73-AF750 (496406, R&D Systems). To exclude non-B cells, the following AF700 conjugated anti-mouse antibodies were combined: F4/80 (BM8), GR-1 (RB6-8C5), CD3 (17A2), CD4 (GK1.5), CD8 (53-6.7), and CD11c (N418) (Biolegend); NKp46 (29A1.4, BD).

A syngeneic decoy (decoy, a recipient-type MHC:peptide tetramer) was used to exclude B cells specific to non-MHC components of the tetramer. Allogeneic tetramer staining was done with the same donor-type MHC molecule conjugated to two separate fluorophores, PE and APC. Double positive (PE^+^/APC^+^) events were donor-reactive MHC antigen-specific allo-B cells, whereas single positive events specific to the fluorophores were excluded. All MHC tetramers were generously provided by the National Institutes of Health (NIH) tetramer core facility which included MHC class I tetramer H-2K^b^ (SIINFEKL) and MHC class II tetramer I-A^b^ (PVSKMRMATPLLMQA) that were conjugated to PE and APC, decoy H-2K^d^-AF488 (SYIPSAEKI), and decoy I-A^d^-BV421 (ISQAVHAAHAEINEAGR).

Flow cytometry samples were run on a Cytek Aurora 5 Laser Cytometer with Spectroflow software (Cytek Biosciences). Cytometry data was analyzed on FlowJo software version 10.8.1 (FlowJo, LLC). Data presentation and statistics were prepared on Prism version 9.5.1 (GraphPad Software, Inc.).

### 2.5 Statistical analysis

Groups were compared using one-way analysis of variance followed by Tukey’s multiple comparison post-test. Significance was reported for comparisons with p<0.05. No significance was found when not indicated with an asterisk. Data were combined from 3-4 independent experiments with 3-4 mice per group except where noted; individual data points represent different mice pooled from repeated experiments unless otherwise noted.

## 3 Results

### 3.1 Alloantibody response

To evaluate if underlying inflammation exacerbates the alloresponse, recipient mice were administered poly(I:C) or LPS four hours prior to non-leukoreduced or leukoreduced platelet transfusion and screened for total and isotype-specific serum anti-donor alloantibodies two weeks later (**Figures 1A–F**). Untreated mice, and mice that received platelet transfusion, poly(I:C), or LPS alone were evaluated as controls. In mice that were administered poly(I:C) or LPS alone, no changes in alloantibodies were detected, but a single non-leukoreduced platelet transfusion significantly increased anti-class I MHC alloantibody responses across all examined isotypes relative to untreated mice (**Figure 2A**). Prior exposure to poly(I:C) followed by platelet transfusion (poly(I:C)/Tx) increased total alloantibody responses whereas prior exposure to LPS followed by platelet transfusion (LPS/Tx) showed a decrease in total alloantibody responses relative to platelet transfusion alone. The elevated total alloantibody in poly(I:C)/Tx was associated with increased IgG2a and IgG3 and decreased IgG1 relative to transfusion alone. The diminished total alloantibody in LPS/Tx was associated with decreased IgM and IgG1 although a downward trend could be observed with all the other isotypes, relative to transfusion alone. Of note, the isotype of greatest magnitude in the LPS/Tx groups was IgM. Responses against class II MHC antibodies were consistent with class I except for subtle differences in IgG2b where prior poly(I:C) exposure elevated responses (**Figure 2B**).

To determine how the presence of WBCs impact the effect of underlying inflammation, the experiment was repeated with leukoreduced platelets (**Figure 3**). The response to leukoreduced platelet transfusion alone was similar to non-leukoreduced, although the magnitude of the responses was smaller, consistent with prior work (38, 39). Moreover, the effect of inflammatory signals on leukoreduced platelets paralleled that of non-leukoreduced platelets where prior poly(I:C) exposure showed trends of elevated responses, whereas prior LPS exposure showed trends of diminished responses for total and isotype-specific alloantibodies. As the effect with leukoreduced platelets were consistent with non-leukoreduced and since leukoreduced platelets were less immunogenic, non-leukoreduced platelets were used as a worse-case model to elicit stronger alloimmunity and examine cellular responses.

### 3.2 MHC tetramer optimization

To understand the mechanism regulating the divergent alloantibody outcomes due to prior exposure to poly(I:C) or LPS, cellular responses of allo-B cells were evaluated. Platelet alloimmunization is driven by mismatched MHC between donor and recipient (in our model, H-2^b^ in B6 mice versus H-2^d^ in Balb/c mice). Although the strategy to identify allo-B cells by utilizing MHC tetramers has been well-established in the cardiac allograft model (48–52), it was unclear whether it could be adopted in our platelet transfusion model due to differences in dose, route of administration, and type of antigenic challenge. To evaluate the feasibility of MHC tetramers in detecting allo-B cells with platelet transfusion, allo-B cell responses were compared among B cell populations of differing specificities; allo-B cells were compared to single-fluorophore specific B cells (APC and PE), B cells specific to the non-MHC components of the tetramers (syngeneic decoy^+^), and the remaining B cell population (**Figure 4A**). Only the allo-B cell population showed significant enrichment in frequency (**Figure 4B**), activation (CD86^+^, **Figure 4C**), antigen experience (IgD^-^, **Figure 4D**), and germinal center (GC) formation (CD95^+^/CD38^-^, **Figure 4E**) after platelet transfusion when evaluated with MHC class I and MHC class II tetramers. Parallel findings were observed in the spleen and lymph node. Thus, MHC tetramers effectively identify allo-B cells in our platelet transfusion model.

### 3.3 Magnitude of the allo-B cell response

While MHC class I is a more abundant source of antigen in our non-leukoreduced platelets (expressed by all platelets and leukocytes), class II antigen is also present (expressed on many blood leukocytes), therefore we evaluated responses to both. An intravenous route of delivery of antigen should engage systemic immunity, not a localized reaction, so spleen and lymph nodes were evaluated for confirmation. Recipient mice were treated as described above, and spleen and lymph nodes were harvested two weeks after platelet transfusion (**Figure 1B**). Allo-B cells were identified as singlets, lymphocytes, live cells, non-B cell^-^/B220^+^ cells, CD19^+^/syngeneic decoy^-^ B cells, and tetramer double-positive events (**Figure 5A**).

The frequency of allo-B cells was consistently low among the non-transfused control groups (untreated, Poly(I:C) only, and LPS only) (**Figures 5B–E**). A single platelet transfusion induced a significant increase of allo-B cells relative to untreated mice (**Figures 5B–E**). Relative to transfusion alone, poly(I:C)/Tx had no effect on the frequency of allo-B cells induced by platelet transfusion (**Figures 5B–E**). Poly(I:C)/Tx showed significantly enhanced allo-B cell frequency when compared to poly(I:C) alone (increased trend only for **Figure 5C**). Strikingly, LPS/Tx showed no significant differences when compared to LPS alone, and importantly, showed reduction in allo-B cell frequency relative to transfusion alone (**Figures 5B, D, E**, decreased trend only for **Figure 5C**). Similar patterns were observed in the spleen and lymph nodes, and against MHC class I and class II, though slightly more pronounced against class II.

### 3.4 Activation and maturation of allo-B cells

Allo-B cell responses were next evaluated for activation by assessing upregulation of CD86, downregulation of IgD (indicating antigen-experience), and formation of GC reactions (CD38^-^/CD95^+^). These populations can be readily identified two weeks after a single platelet transfusion (**Figures 6A**, **7A**, **8A**). In the non-transfused control groups, little to no activation of allo-B cells was observed (**Figures 6**–**8**), except for the LPS only group which showed increased IgD downregulation relative to untreated mice in the spleen (**Figures 7B, D**, trend only in D). Two weeks after a single allogeneic platelet transfusion, the frequency of activated, antigen-experienced, and GC allo-B cells were increased compared with the untreated group (**Figures 6**–**8**). Prior exposure to poly(I:C) in the poly(I:C)/Tx group did not induce more allo-B cell activation, antigen-experience, or germinal center reactions when compared to platelet transfusion alone (**Figures 6**–**8**). This group did show significant increases in frequencies of activated, antigen-experienced, and GC allo-B cells relative to the poly(I:C) only group (**Figures 6**–**8**, increased trend only in **Figure 8C**). Conversely, frequencies of activated, antigen-experienced, and GC allo-B cells for the LPS/Tx group were not significantly different than the LPS only group but were significantly reduced relative to transfusion alone for responses in the spleen (**Figures 6**–**8B, D**). In the lymph node, the allo-B cell responses for the LPS/Tx group were significantly increased relative to LPS alone but were still significantly reduced relative to transfusion alone (**Figures 6**-**8C, E**, reduced trend only observed for **Figures 6C**, **8C**). Generally, while trends were similar, responses in the lymph node were more pronounced than in the spleen, and responses against MHC class II were more pronounced than MHC class I (**Figures 6**–**8**). The levels of CD86 expression were evaluated in activated CD86^+^ allo-B cells by MFI, and no differences were observed when transfusion alone was compared to poly(I:C)/Tx but expression levels trended downwards in the lymph node and were significantly reduced in the spleen when transfusion alone was compared to LPS/Tx (data not shown), consistent with frequency data.

### 3.5 Marginal zone B cell response

To evaluate whether marginal zone (MZ) B cells played an important role in our model, MZ B cells were identified as CD93^-^ (mature), CD95^-^/CD38^+^ (GC-negative), and IgM^+^/CD23^-^ (MZ), and their frequencies within the total B cell and allo-B cell populations were assessed (**Figure 9A**). No significant differences were observed after transfusion for total MZ B cells in the spleen and lymph node (**Figures 9B, C**). Groups treated with LPS alone showed a significant increase in total MZ B cells (trend only in lymph node, **Figures 9B, C**), when compared to untreated or transfused only groups. However, this appears independent of transfusion, and others have observed LPS administration directly stimulates MZ B cells (53–55). While no significant increases in MZ allo-B cells were observed after transfusion for the respective pairs, a significant increase was seen for the Poly(I:C)/Tx group relative to the untreated and transfusion only groups (**Figures 9D, E**). Like total MZ B cells, MZ allo-B cells were non-specifically induced by LPS treatment (**Figures 9D, E**). No differences were observed in activation and IgD downregulation after transfusion in total MZ and MZ allo-B cells (data not shown). Lymph node MZ allo-B cells were too rare to quantify (data not shown).

### 3.6 Memory B cell responses

We next evaluated the formation of memory B cells within the allo-B cell population. Total memory B cells were defined as non-GC, antigen-experienced, mature allo-B cells (**Figure 10A**). This population was further delineated into phenotypically and functionally distinct memory subsets by the expression of CD80 and PD-L2 (**Figure 10A**) (56). Functionally, CD80^-^/PD-L2^-^ double-negative memory B cells readily seed GC reactions, whereas CD80^+^/PD-L2^+^ double-positive memory B cells are poised to differentiate into antibody-secreting cells (57). Although total allo-memory B cells were readily measured in the spleen and lymph node (**Figures 10B–E**), these events are rare, and the subset double-negative and double-positive memory B cell events were too few to quantify in the lymph node (data not shown). As seen with activation, allo-memory B cell responses were more pronounced in the lymph node compared with the spleen, and against MHC class II compared with class I (**Figure 10**). While Poly(I:C) alone did not alter allo-memory B cell responses (**Figures 10**), the LPS alone group had increased total allo-memory B cell responses in the spleen (**Figures 10B, D**). Compared to untreated mice, a single allogeneic transfusion showed an increase in total allo-memory B cells but was significant only in the lymph node against MHC class II (**Figures 10B–E**). The poly(I:C)/Tx group showed significantly elevated responses relative to poly(I:C) alone for total allo-memory B cells (**Figures 10C–E**, no difference in **Figure 10B**), and these responses trended higher than the transfusion alone group (**Figures 10C–E**, significant in 10C). The LPS/Tx group showed no differences in total allo-memory B cells relative to LPS only for responses against MHC class I (**Figures 10B, C**) and showed an increased trend for class II responses (**Figures 10D, E**). Relative to transfusion alone, total allo-memory B cells in the LPS/Tx group was elevated in the spleen (**Figures 10B, D**, significant for 10B), and trended down in the lymph node (**Figures 10C, E**). For allo-memory B cell responses against MHC class II, the transfusion alone and poly(I:C)/Tx groups skewed towards the CD80^+^/PD-L2^+^ double-positive subset (**Figures 10H, I**). In contrast, the non-specific responses against LPS (LPS only and LPS/Tx groups) skewed more towards the PDL2^-^/CD80^-^ double-negative subset (**Figures 10F–I**).

## 4 Discussion

In this study, we have demonstrated that contrary to our hypothesis, not all inflammatory states enhance the alloresponse, with LPS inhibiting the response to allogeneic platelet transfusion. Using a novel, and highly sensitive tetramer staining approach, we characterized the primary B cell response to allogeneic platelet transfusion. We demonstrated that the reduction in alloantibody responses seen with LPS were accompanied by reduced activation of allo-B cells, reduced differentiation into GC B cells, and reduced PDL2^+^/CD80^+^ memory B cells. Poly(I:C), on the other hand, enhanced the alloantibody response and was associated with more robust differentiation into memory B cells in the lymph nodes.

Here we used a novel tetramer staining strategy along with our established platelet transfusion model to assess the antigen-specific B cell response to transfusion. The use of MHC tetramers to track the behavior of allo-B cells has been employed in the cardiac allograft model in mice (48–52, 58), and similarly human leukocyte antigen (HLA) tetramers were used to examine allo-B cells in clinical samples (52, 59), but this strategy had not yet been adopted in the field of transfusion medicine. We demonstrated for the first time that a single allogeneic platelet transfusion elicits robust responses from the rare, endogenous donor-reactive allogeneic MHC antigen-responsive B cell population, with increased activation, GC and memory formation. Memory responses appeared to be slightly skewed towards the CD80^+^/PD-L2^+^ double-positive subset, suggesting priming towards efficient future antibody production (57). In contrast with depletion studies from other groups showing a critical role for MZ B cells in RBC and platelet alloimmunization (60–62), we did not see much of a shift in the MZ allo-B cell population. This difference may be due in part to experimental approach, i.e. depletion versus examining endogenous responses at a set time which may have missed the peak MZ response, and/or due to differences in cell type (RBCs and leukoreduced platelets versus non-leukoreduced platelets).

Until now, there has been a paucity of information on the effect of underlying inflammation on platelet alloimmunization. Our results on the antibody response to platelet transfusion are, however, consistent with models of inflammation in RBC alloimmunization, where poly(I:C) enhanced and LPS inhibited humoral alloimmunization (23, 26, 63, 64). Within this model, poly(I:C) was shown to enhance RBC alloantigen processing by dendritic cells leading to increased CD4^+^ T cell activation (24). Conversely, prior exposure with LPS, consistent with the timing of LPS exposure in our model, inhibits dendritic cells and prevents them from processing RBC alloantigen which abrogates CD4^+^ T cell help needed for alloimmune responses (65). These data are also consistent with clinical observations of increased trends of RBC alloimmunization among patients experiencing disseminated viral disorders, and potentially decreased RBC alloimmunization among patients suffering from Gram-negative bacteremia (27).

Allo-B cell responses to platelet transfusion were significantly altered by poly(I:C) and LPS. The poly(I:C)/Tx group had allo-B cell responses that were overall similar when compared to transfusion alone, with the exception of more robust memory B cell responses in the lymph node. Like with transfusion alone, the memory cells were skewed towards the CD80^+^/PD-L2^+^ double-positive subset, which may make secondary antibody responses more efficient. The elevated primary antibody responses seen with poly(I:C)/Tx are striking and likely driven by generation of plasma cells, although we were unable to directly access this due to the rarity of plasma cell events at the timepoints analyzed. The elevated total alloantibody responses due to poly(I:C)/Tx were associated with decreased IgG1 and increased T_H1_-biased IgG subclasses IgG2a and IgG3 relative to transfusion alone. The IgG1 isotype is thought to be inhibitory to IgG3 aggregation which suggests inflammatory IgG3 responses were less regulated (66). This is consistent with how Poly(I:C) is known to induce viral-like responses and skewing immunity towards T_H1_ responses (67, 68). How poly(I:C) modulates platelet alloimmunization is unclear. Perhaps poly(I:C) engages toll-like receptor (TLR)3 which activates innate immunity that leads to anti-viral responses, which may promote increased T_H1_-biased alloantibody responses (67, 69). Human plasma cells express TLR3 and increase production of IgM and IgG upon TLR stimulation (70). Poly(I:C) also induces immunity through TLR3 independent pathways. In RBC alloimmunization, poly(I:C) enhances alloimmunization via MAVS pathway rather than agonizing TLR3 (71).

Unlike the heightened alloantibody response due to poly(I:C), LPS had a striking and opposing effect. LPS is known to induce T_H1_-like cytokine responses and favors IgG3 class switch (72, 73). However, prior exposure to LPS, a TLR4 agonist, dampened all alloantibody isotype responses, specifically IgM and IgG1, and all associated allo-B cell responses examined, which suggests LPS abrogates B cell activation in our model. In fact, of the isotypes, IgM expression was the greatest in magnitude for LPS/Tx. While counterintuitive given the established role of LPS in inflammation and bystander activation (32, 33), it is consistent with clinical reports of transient immune paralysis or “LPS tolerance” due to sepsis-induced inflammation (74–77).

As expected, LPS administration alone was sufficient to cause non-specific induction of immature/transitional (data not shown), MZ, and memory allo-B cells (78–80). We did observe an expansion of IgD^-^ allo-B cells with LPS alone, which are likely IgD^low^ transitional B cells. They are unlikely activated B cells given their lack of CD86 or germinal center markers.

While trends of anti-MHC antibody responses were consistent between class I and class II, the magnitude of total anti-MHC antibody responses were ~20x greater than untreated for class II whereas they were ~10x greater for class I, although direct comparisons cannot be made. Allo-B cell responses against MHC class II were generally more robust and greater in magnitude than those against MHC class I. As MHC class I is expressed on the surface of both platelets and WBCs, whereas MHC class II is found only on a subset of WBCs, the density of exposure to MHC class I antigens from a platelet product was presumably greater (5). Despite the lower exposure, MHC class II antigens were comparatively more immunogenic than class I antigens in responses in the spleen and lymph node. One possible explanation is that the MHC class II alloantigens are directly presented to CD4^+^ T cells, leading to more effective T cell help for the B cell response. Minimizing these potent MHC class II responses may explain mechanistically one of the benefits of the use of leukoreduction for platelet transfusion.

Responses in the spleen mirrored those in the lymph node although surprisingly, many responses were more robust in the lymph node. This was in stark contrast to the RBC model, where no RBC consumption by macrophages or dendritic cells were observed in the lymph node and where the spleen was shown to be an essential site for alloimmunization (24, 61, 65, 81, 82). While this apparent conflict could be due to differences in platelet and RBC uptake, a study using leukoreduced platelets suggested allogeneic-specific CD4^+^ T cell reactions occur in the spleen and not the lymph node (83). Taken together, this suggests that the donor lymphocytes may play an important role in platelet alloimmunization, either through a different cellular source of antigen or through the addition of MHC class II antigens. Additionally, our MHC tetramer staining strategy may have provided greater sensitivity to observe rare endogenous allo-B cell events that may not have been previously appreciated. Alternatively, alloimmunity may initiate in the spleen and subsequently seed downstream responses in the lymph node.

While our study has demonstrated that different inflammatory signals can alter the alloresponse, some limitations should be considered. While our murine models enabled a controlled approach, confirmation will be needed clinically to determine if similar responses are observed with human transfusions. We also utilized non-leukoreduced platelets to examine the cellular allo-B cell responses in our study as they generate more potent immune responses. Although a similar effect of inflammatory signals on alloantibodies was observed between anti-MHC class I and class II responses with non-leukoreduced platelets and anti-MHC class I responses with leukoreduced platelets, it is possible that the lower antigen dose environment or other platelet-specific attributes of leukoreduced platelets could alter allo-B cell responses. Finally, while LPS and poly(I:C) deliver some of the critical innate immune signals seen in gram-negative bacterial infection and viral infection, respectively, an active infection would introduce additional complexity and may further alter alloimmunization outcomes.

In conclusion, these data demonstrate that not all inflammatory environments enhance bystander responses. Prior exposure to LPS on gram-negative bacteria may, in fact, curtail platelet alloimmunization. Together, these observations may inform clinical practice to better manage risk for platelet alloimmunization.

## Data availability statement

The raw data supporting the conclusions of this article will be made available by the authors, without undue reservation.

## Ethics statement

The animal study was approved by Labcorp Early Developmental Laboratories Inc. The study was conducted in accordance with the local legislation and institutional requirements.

## Author contributions

JT: Data curation, Formal analysis, Investigation, Methodology, Project administration, Resources, Software, Supervision, Validation, Visualization, Writing – original draft, Writing – review & editing. MM: Data curation, Writing – review & editing, Investigation, Methodology, Project administration, Resources. BG: Data curation, Investigation, Methodology, Project administration, Writing – review & editing. OD: Data curation, Investigation, Methodology, Project administration, Writing – review & editing. MT: Formal analysis, Supervision, Validation, Writing – review & editing. RJ: Conceptualization, Data curation, Funding acquisition, Investigation, Methodology, Project administration, Resources, Software, Supervision, Validation, Visualization, Writing – review & editing.
